# Exploratory Evaluation of Susceptibility Patterns of Clinical Isolates of *Acinetobacter baumannii* Towards Polymyxins and Different Cationic Antimicrobials

**DOI:** 10.3390/antibiotics15080726

**Published:** 2026-07-27

**Authors:** Shakeel Shahzad, Mark D. P. Willcox, Bushra Jamil, Binod Rayamajhee, Muhammad Yasir

**Affiliations:** 1 School of Optometry and Vision Science, Faculty of Medicine and Health, University of New South Wales UNSW, Sydney 2052, Australia; 2 BJ Micro Lab Private Limited, Rawalpindi 46222, Pakistan; 3 Department of Allied Medical and Health Sciences, Sir Syed CASE University, Islamabad 44000, Pakistan; 4 Faculty of Medicine, Health and Human Sciences, Macquarie University, Sydney 2113, Australia

**Keywords:** cationic antimicrobials, clinical isolates, *Acinetobacter baumannii*, polymyxin, resistance

## Abstract

Background: Carbapenem-resistant *Acinetobacter baumannii* is a critical priority pathogen of the *Acinetobacter calcoaceticus*-*Acinetobacter baumannii* Complex (*Acb* Complex) characterized by extensive multidrug resistance, leading to increased reliance on last-line polymyxins and widespread use of cationic disinfectants in healthcare settings. However, shared membrane-targeting mechanisms raise concerns regarding potential cross-resistance between disinfectants, polymyxins, and cationic antimicrobial peptides. Methods: A diverse panel of clinical *A. baumannii* isolates from Australia (*n* = 9), Pakistan (*n* = 15), and Switzerland (*n* = 3), and standard strains (*n* = 3), including polymyxin-resistant laboratory and clinical mutant strains (*n* = 4), was used. Minimum inhibitory concentrations (MICs) of polymyxin B, colistin, three cationic disinfectants (polyquaternium-1; PQ-1), polyhexamethylene biguanide (PHMB), and chlorhexidine (CHX), and four Antimicrobial peptides (AMPS: LL-37, melimine, Mel4, and lactoferricin) were determined using broth microdilution. Correlations between MICs were assessed using Spearman analysis, and differences between polymyxin-resistant and -susceptible isolates were analyzed statistically. Results: Most isolates were susceptible to polymyxins. The MIC data confirmed that one mutant strain, *A. baumannii* 19606 (R), and three clinical isolates displayed high-level polymyxin B and colistin resistance. PQ-1 exhibited the greatest antibacterial activity among disinfectants, while LL-37 showed the lowest and most consistent MICs among AMPs; lactoferricin was largely inactive. Within this exploratory strain collection, polymyxin-resistant isolates exhibited lower MICs to PQ-1 and PHMB, indicating collateral susceptibility. Correlation analysis revealed significant negative correlations between polymyxin susceptibility and disinfectant MICs (colistin–PQ-1 (r = −0.67 and *p* = 0.0001), colistin–PHMB (r = −0.44 and *p* = 0.05), and polymyxin B–PHMB (r = −0.48 and *p* = 0.005), whereas no consistent correlation was observed between polymyxins and AMPs, including LL-37. A moderate positive correlation was identified between colistin and Mel4 activity (r = 0.45 and *p* = 0.05). Conclusions: These findings indicate diverse susceptibility patterns of *A. baumannii* to cationic antimicrobials. Within this exploratory strain collection, polymyxin resistance was not associated with reduced susceptibility to disinfectants or most antimicrobial peptides and was instead associated with lower MICs to certain disinfectants. No evidence of cross-resistance between polymyxins and LL-37 was observed under the conditions tested, suggesting that polymyxin resistance does not necessarily confer reduced in vitro susceptibility to this host defense peptide. Given the small number of polymyxin-resistant isolates, these findings should be considered hypothesis-generating and require confirmation in larger collections of genetically characterized strains. Further mechanistic studies are needed to determine the biological basis and generalizability of these observed susceptibility patterns.

## 1. Introduction

The *Acinetobacter* genus is diverse, comprising oxidase-negative, nonpigmented, Gram-negative *coccobacilli*. With over 50 species identified [1] most are environmental and non-pathogenic. The species most frequently linked to infections are *A. baumannii*, followed by *A. calcoaceticus* and *A. lwoffii* [2], then other species including *A. haemolyticus*, *A. johnsonii*, *A. junii*, and *A. ursingii* [3,4,5,6,7,8,9]. *A. baumannii* is the most virulent species among them [10].

Infections caused by *Acinetobacter* spp. became more widespread in the 1960s and 1970s, coinciding with the growing use of intensive care techniques [11,12], mechanical ventilation, central venous and urinary catheterization, and associated antibacterial therapies [13,14,15,16]. Analysis of data from 2018 to 2020 found *Acinetobacter* spp. accounted for 1.2% of all healthcare-associated infections in the USA [17]. There are an estimated 1 million cases globally each year [18]. Similar infection rates are observed in Intensive Care Units (ICUs) across Europe and Latin America [19,20,21,22,23]. In some Asian and Latin American countries, *Acinetobacter baumannii* ranks among the top three causes of bacteraemia and nosocomial pneumonia [24,25,26,27,28].

Carbapenem-resistant *Acinetobacter baumannii* is ranked as a top priority pathogen of critical importance by the World Health Organization for which new antimicrobials are urgently required [29]. It has increasing resistance to all traditional antibiotics, with up to 90% of strains being resistant to cephalosporins, carbapenem, fluoroquinolones, and other β-lactams [30,31]. Furthermore, multidrug resistance has been reported in up to 99.8% of carbapenem-resistant *A. baumannii* [32,33]. Infection with carbapenem-resistant strains results in higher in-hospital mortality [17]. Polymyxins are becoming more widely used to treat *Acinetobacter* infections due to this increase in resistance to traditional antibiotics. However, resistance to polymyxins is also increasing [34,35] with 21% of strains being resistant to colistin in one study [30]. *A. baumannii* can become resistant to polymyxins via a variety of mechanisms, including addition of phosphoethanolamine to its outer membrane, loss of lipopolysaccharide from its outer membrane, loss of outer membrane proteins such as OmpA, and expression of efflux pumps [36,37].

Due to the potential for cross-contamination, disinfection of hospital surfaces and appliances is common. Cationic disinfectants such as chlorhexidine (CHX), polyhexamethylene biguanide (PHMB) and polyquaternium-1 (PQ-1) are widely used for disinfection purposes in health care settings [38,39,40]. However, excessive or improper use of disinfectants can contribute to bacterial resistance and promote cross-resistance to antibiotics, thereby worsening the global antimicrobial resistance crisis [41]. Common disinfectants such as CHX may trigger adaptive bacterial mechanisms such as efflux pump activation, biofilm formation, membrane alterations, stress-response pathways, and horizontal gene transfer [42]. These adaptations can increase bacterial tolerance not only to disinfectants but also to multiple antibiotics, leading to multidrug-resistant pathogens [42]. There is the potential for resistance to cationic disinfectants to affect the susceptibility to polymyxins [43]. 

Cationic antimicrobial peptides are possible alternatives to new antibiotics or disinfecting agents that are being developed. They typically consist of short chains of amino acids and their net positive charge at physiological pH is usually due to the presence of lysine or arginine [44]. This positive charge is critical for their interaction with microbial cell membranes, which are often negatively charged [45]. The naturally occurring human cathelicidin LL-37 and the chimeric AMP melimine have been studied for their antimicrobial activity [46,47,48]. Use of LL-37 can improve polymicrobial wound infection in clinical trials [49,50], and a modified version of melimine, Mel4, successfully completed a Phase III clinical trial as an antimicrobial contact lens coating [51]. The changes that occur in membranes to develop resistance to polymyxins and cationic disinfectants can also affect sensitivity to antimicrobial peptides [52], although this is not always the case [53,54].

This research was designed to determine the susceptibility pattern of clinical isolates of *A. baumannii* against disinfectants, polymyxins and cationic antimicrobial peptides, and to evaluate whether resistance to disinfectants correlated to resistance to polymyxins, and whether resistance to polymyxins was correlated with resistance to antimicrobial peptides. The hypotheses being tested were (1) resistance to disinfectants would correlate with resistance to polymyxin antibiotics, and (2) resistance to polymyxin antibiotics would correlate with resistance to naturally occurring and synthetic antimicrobial peptides.

## 2. Results

### 2.1. Confirmation of Strain Identity

All strains were confirmed as *A. baumannii* (Table 1). Apart from strains ATCC 19606, ATCC 19606 (R), Ab08, Ab39 and Ab61, other strains were not selected on the basis of their resistance to polymyxins or any of the other antimicrobials being studied.

### 2.2. Minimum Inhibitory Concentration of Cationic Antimicrobials

The results demonstrate diverse patterns of susceptibility toward different tested cationic antimicrobials. With the exception of four polymyxin-resistant strains (ATCC 19606 (R), Ab08, Ab39 and Ab 61), all other tested strains were susceptible to polymyxin B (1–2 μg/mL) and colistin (0.25–2 μg/mL). The MICs to polymyxin B for the resistant strains ranged from 16 μg/mL for Ab08 to 1250 μg/mL for ATCC 19606 (R) (Table 2). For the disinfectants, PQ-1 was the most effective with a median MIC of 18.75 μg/mL, ranging from 6.3 to 50 μg/mL (Table 2). The median MICs for PHMB and CHX were the same, at 25 μg/mL, although there was a slightly greater range for PHMB of 3.13 to 50 μg/mL (Table 2).

For the antimicrobial peptides, LL-37 gave the most consistently low MICs, with a median of 62.5 μg/mL, ranging from 31.3 to 125 μg/mL (Table 2). This was followed by Mel4 and melimine, with median MICs of 125 μg/mL, but with a slightly greater range from 15.6 to 250 μg/mL for Mel4 (Table 2). Lactoferricin was virtually inactive against the strains tested, with MICs > 500 μg/mL (Table 2).

Overall, Australian and Pakistani isolates had very similar median MICs to each antimicrobial, with the exception of Mel4, where the median MIC for Pakistani isolates was 250 μg/mL compared to 62.5 μg/mL for Australian isolates.

When the MICs to Mel4, melimine, LL-37, PQ-1, PHMB, CHX of the polymyxin resistance isolates (ATCC 19606 (R), Ab08, Ab39, Ab61) were compared with the polymyxin susceptible strains, there were markedly lower MICs to PQ-1 (median 6.3 vs. 25; *p* = 0.01) and PHMB (median 3.1 vs. 25; *p* = 0.003), with a trend of the same difference occurring with CHX (15.6 vs. 25; *p* = 0.08) consistent with collateral susceptibility, although confirmation in larger collections is required. In contrast, susceptibility to antimicrobial peptides (Mel4, melimine, LL-37) showed no consistent directional change.

### 2.3. Correlation Analysis of MICs

**Figure 1:**
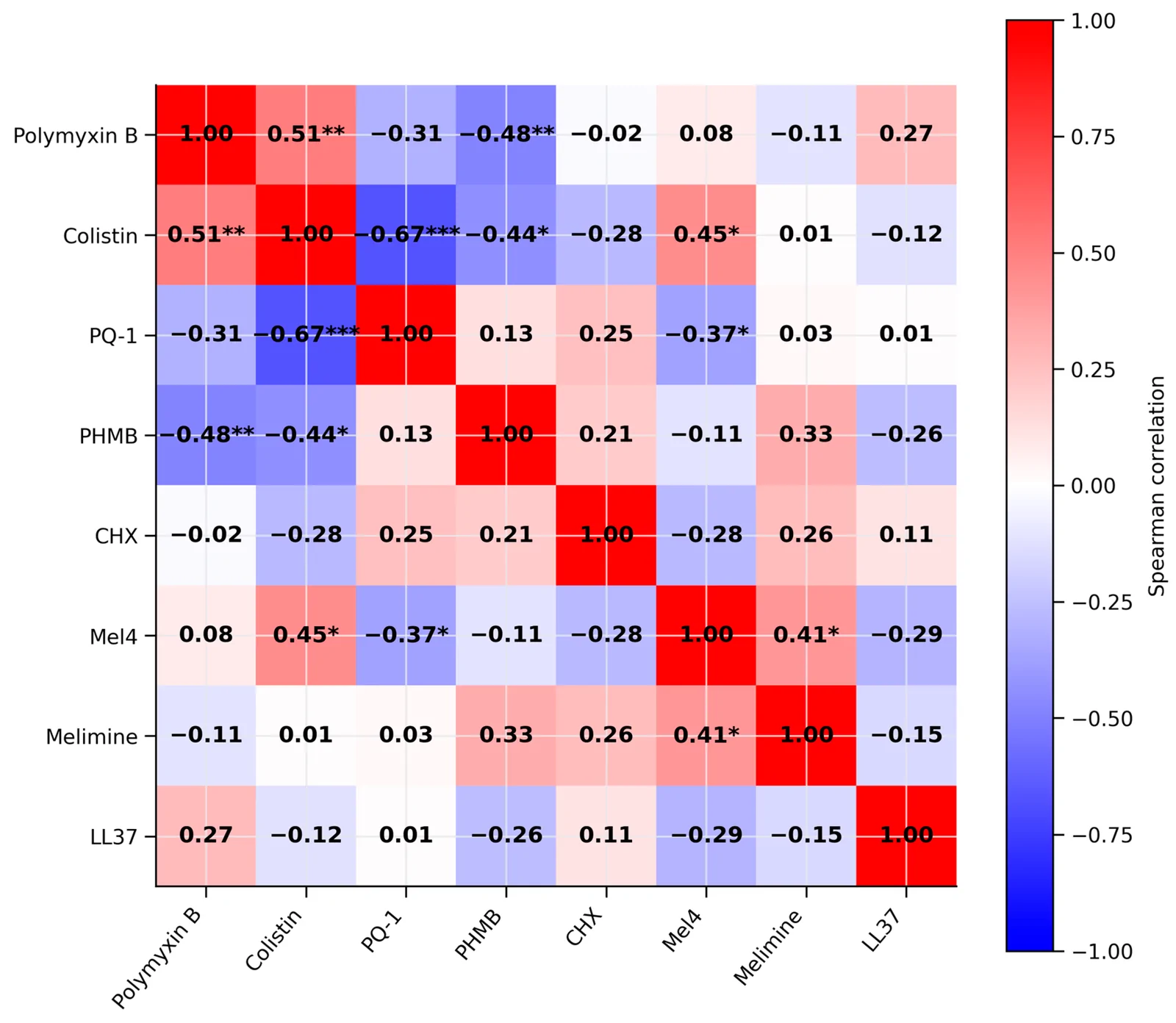
Heatmap of antimicrobial agents showing the relationships between their activity profiles. Each cell represents the Spearman correlation coefficient (r) between a pair of compounds, with values ranging from −1.00 (strong negative correlation) to +1.00 (strong positive correlation). Dark red indicates positive correlations, white represents no correlation, and blue reflects negative correlations. ***, *p* = 0.0001; **, *p* < 0.005; *, *p* ≤ 0.05.

Within the antimicrobial groups, polymyxin B and colistin showed a strong positive correlation (r = 0.51; *p* = 0.0041; Figure 1). Mel4 and colistin demonstrated the next strongest correlation (r = 0.45; *p* = 0.05), followed by Mel4 and melimine (r = 0.41; *p* = 0.0258). On the other hand, colistin was significantly negatively correlated with PQ-1 (r = −0.67; *p* = 0.0001) and PHMB (r = −0.44; *p* = 0.05). Similarly, MICs of polymyxin B were significantly negatively correlated with PHMB MICs (r = −0.48; *p* = 0.005). In contrast, LL-37 susceptibility was not significantly correlated with any of the other antimicrobial tests.

## 3. Discussion

The present study was designed to evaluate the pattern of susceptibility among *A. baumannii* strains against different cationic antimicrobials and to evaluate whether high MICs to disinfectants correlate to resistance to polymyxins or MICs to cationic antimicrobial peptides. The impetus for this research was the use of disinfectants to control the spread of *A. baumannii* and the increasing isolation of polymyxin-resistant strains. The hypothesis was that resistance to disinfectants might cross-react to resistance to polymyxins, as these can act in similar ways on the membrane of bacteria. Furthermore, mechanisms of resistance to disinfectants such as efflux pump activation and membrane alterations [42] are also used by *A. baumannii* to become resistant to polymyxins [34].

The results of this study show that the hypothesis was not proven, and resistance to polymyxin in *A. baumannii* negatively correlated with resistance to disinfectants (especially PQ-1 and PHMB). Whilst the antibacterial mechanism of action of PHMB has been traditionally thought to be via membrane disruption, recent studies have shown that whilst PHMB can attach to Gram-negative bacterial membranes, this does not result in pore formation (often a key mechanism for antibacterial activity), but it instead penetrates through the membranes and interacts with intracellular DNA, which is likely the antibacterial mode of action [56,57]. Similarly, the quaternary amine compound benzalkonium chloride can have two modes of action against *A. baumannii* [58]. At high concentrations, benzalkonium chloride acts on the bacterial membrane, but at lower concentrations it can induce protein aggregation, and this can lead to cell death [58]. This may also occur for polyquats such as PQ-1. The polymyxin antibiotics appear to have predominant modes of action on the outer membrane of *A. baumannii* and other Gram-negative bacteria, although they may also act intracellularly by inhibiting respiratory enzymes and triggering production of reactive oxygen species [59,60]. These differences may at least partially account for the lack of correlation between the MICs for the polymyxins and disinfectants. Reinforcing this, the polymyxin-resistant mutant *A. baumannii* ATCC 19606 (R) with complete loss of LPS production [55], with MICs of 1250 μg/mL and >2500 μg/mL against polymyxin B and colistin, respectively, showed high sensitivity to these disinfectants. A previous study found that loss of LPS caused a 4-fold reduction in MIC of CHX [61].

This study also evaluated whether high MICs to disinfectants or resistance to polymyxins resulted in high MICs to antimicrobial peptides [62]. The AMPs chosen for study were two naturally occurring AMPs, LL-37 (the only human cathelicidin) and lactoferricin (a small peptide from lactoferrin), as well as the synthetic AMPs melimine and Mel4. Should there have been a correlation, it would mean that resistance to polymyxins might have large impacts on the pathogenesis of disease caused by *A. baumannii*. Epithelial cells upregulate AMPs during *A. baumannii* infection [63] and AMPs such as LL-37 are key components of the innate defense system [64]. Furthermore, the AMPs melimine and Mel4 are being examined as potential new therapeutic or prophylactic agents [65,66,67], and so cross-resistance might curtail this research and development. Previous studies have determined that polymyxin resistance in *A. baumannii* is mediated by several factors, including phosphoethanolamine addition to lipopolysaccharides in the outer membrane, loss of lipopolysaccharide from the outer membrane, and overexpression of efflux pumps [34,36,55]. These same mechanisms can confer resistance to antimicrobial peptides such as LL-37 [46]. Indeed, clinical use of polymyxin can induce cross-resistance to host antimicrobial peptides in *A. baumannii* [43].

However, the current study found no statistically significant evidence for cross-resistance between the naturally occurring LL-37 and polymyxins. This implies a lack of effect of polymyxin resistance, especially loss of the outer membrane, which is the known mechanism of resistance of strain ATCC 19600 (R), on susceptibility to LL-37. There was a significant correlation between colistin resistance and MIC to Mel4, implying the possibility of similar mechanisms being involved. However, this correlation did not hold for polymyxin B, which is structurally similar to colistin. Further research is needed to identify if any known polymyxin resistance mechanisms can confer cross-resistance to Mel4, and why these do not correlate with resistance to polymyxin B or MICs to melimine (the parent peptide of Mel4). The correlation between MIC to Mel4 and PQ-1 is intriguing, suggesting some cross-resistance, and this should also be followed up in future experiments. These future experiments could mutate susceptible strains with genes known to be involved in polymyxin, disinfectant and antimicrobial peptides and determine which ones confer cross-resistance. The Mann–Whitney comparison between the polymyxin-resistant and susceptible groups is limited by the very small resistant group, and future studies should increase the number of these strains used. Furthermore, clinical isolates were selected based on polymyxin susceptibility phenotype rather than known resistance mechanism. Consequently, the polymyxin-resistant group likely comprises isolates with diverse genetic determinants of resistance. The study therefore examines the association between polymyxin resistance phenotype and susceptibility to LL-37 and chlorhexidine, rather than the effects of specific resistance mechanisms. Future studies incorporating genomic characterization are needed to determine whether observed cross-resistance patterns are mechanism-dependent.

## 4. Materials and Methods

### 4.1. Collection and Preservation of Bacterial Strains

Clinical isolates of *A. baumannii* were collected from Australia (Liping Li, Macquarie University, Sydney, NSW, Australia), Pakistan (Bushra Jamil, Sir Syed CASE University, Islamabad, Pakistan) and Switzerland (Stefano Mancini, Institute of Medical Microbiology, University Zurich, Zürich, Switzerland). This study included the polymyxin-resistant mutant *A. baumannii* ATCC 19606 (R), which has a complete loss of LPS production due to a mutation in *lpx* genes [55]. All the strains were preserved in 25% glycerol and stored at −80 °C at the microbial culture collection of the School of Optometry and Vision Science, Faculty of Medicine and Health, UNSW Sydney.

### 4.2. Confirmation of Strain Identities by MALDI-TOF MS

Identity of strains was confirmed by MALDI-TOF MS (Matrix-assisted laser desorption/ionization time-of-flight mass spectrometry). Briefly, strains were cultured on blood agar overnight and protein extraction was done according to the MALDI Biotyper^®^ Protocol Guideline [68,69] with 300 μL of sterile Milli-Q water. After air-drying the pellet at room temperature, 25 μL of 70% aqueous formic acid was added and mixed, followed by addition of 25 μL 100% acetonitrile and mixing. The mixture was then centrifuged for 2 min at 13,000 rpm and 1 μL of the supernatant was deposited onto a MALDI target plate and allowed to dry at room temperature. A 1 μL aliquot of Bruker Bacterial Test Standard (Bruker, Billerica, MA, USA) was transferred to a MALDI target plate and allowed to dry. After drying, 1 μL of HCCA (α-Cyano-4-hydroxycinnamic acid) matrix solution was added to each sample and standard. Identification was done by using a Bruker ultrafleXtreme MALDI-TOF/TOF biotyper system.

### 4.3. Determination of Minimum Inhibitory Concentrations of the Antimicrobials

Two cationic polypeptide antibiotics, polymyxin B (PMB) (Fluka Chemie GmbH, Buchs, Switzerland) and colistin sulfate (Polymyxin E; Sigma-Aldrich, Burlington, MA, USA), were used. Four cationic antimicrobial peptides (lactoferricin, LL-37, melimine, and Mel4) were synthesized by solid-state synthesis and purchased from AusPep Peptide Company (Tullamarine, VIC, Australia) with a purity of ≥90%. The cationic disinfectants PQ-1 (Sigma-Aldrich), PHMB (Biosynth, Gardner, MA, USA) and CHX (Sigma-Aldrich) were also used.

Minimum inhibitory concentration (MIC) of the cationic antimicrobials was determined by broth micro-dilution assay [70] according to the CLSI guideline [71]. Strain ATCC 19606 was used as a quality control for polymyxin resistance, as its MIC, using CLSI guidelines, had been previously published [72]. For ATCC 19606 (R), stock solutions of 5000 μg/mL in sterile water were prepared for polymyxin B and colistin; for all other strains, stock solutions of 1024 μg/mL were prepared. For PQ-1, PHMB, and CHX, stock solutions of 200 μg/mL were prepared in sterile distilled water. For the AMPs, standard solutions of 1mg/mL were made in sterile distilled water. Cation-adjusted Mueller–Hinton broth (Ca-MHB; Oxoid, Basingstoke, Hampshire, UK) was used as described previously by Wiegand et al. (2008) [73]. Briefly, 100 μL of each antimicrobial was diluted 2-fold in 100 μL Ca-MHB, then serially diluted in Ca-MHB. A standard bacterial inoculum was prepared from the overnight culture by adjusting it to 0.5 McFarland standard (~1.5 × 10^8^ colony forming units (CFU)/mL), followed by further dilution to achieve a final concentration of approximately 1.5 × 10^6^ CFU/mL. Next, 100 μL of bacterial cells were added to each well, plates were incubated for 24 h, and MIC was determined as inhibition of visible growth. All experiments were performed in triplicate, and there were no differences between replicates.

### 4.4. Statistical Analysis

Correlation analysis among MICs of different cationic antimicrobials was determined by GraphPad Prism software (version 11.02.2. GraphPad Software, San Diego, CA, USA) by determining the Spearman correlation coefficient from MIC data after log_2_-transforming the MIC. Mann–Whitney U test was performed to determine whether differences in the median MICs of polymyxin-resistant and susceptible strains to Mel4, melimine, LL-37, PQ-1, PMHB, and CHX were statistically different. Where MIC data were greater than the highest concentration tested, i.e., >500 or >2500, these were converted to 500 or 2500, respectively.

## 5. Conclusions

*A. baumannii* strains demonstrate diverse patterns of susceptibility to cationic antimicrobial peptides and disinfectants. One research question being tested was whether high MICs to disinfectants that can be used to clean surfaces to reduce the spread of *Acinetobacter* spp. were correlated to resistance (high MICs) to polymyxin antibiotics, which are antibiotics of last resort to treat *A. baumannii* infections. The study found an inverse correlation, implying that disinfectant resistance may not result in resistance to polymyxins. The second hypothesis being tested was whether resistance to polymyxins was correlated to resistance to antimicrobial peptides involved in defense against *A. baumannii* that are being developed as new therapeutics or prophylactic agents. The lack of correlation between MIC for LL-37 and polymyxins in this strain collection implies no cross-resistance.

This study demonstrated diverse susceptibility patterns among *A. baumannii* isolates toward polymyxins, disinfectants and antimicrobial peptides. Within this strain collection, polymyxin resistance was not associated with reduced susceptibility to the disinfectants PQ-1 or PHMB and was not associated with reduced susceptibility to LL-37. These findings suggest that cross-resistance between polymyxins and these membrane-active compounds was not evident under the conditions examined. However, because the polymyxin-resistant subgroup was intentionally enriched and comprised only four isolates, these observations should be regarded as exploratory and hypothesis-generating. Larger studies incorporating genetically characterized resistant isolates and mechanistic analyses will be required to determine whether these susceptibility patterns are broadly generalizable and to define the biological basis of the observed collateral susceptibility.

## Figures and Tables

**Table 1:** The strains used in the current study.

Strain Code	Origin (Disease, Body Site, Country, Year of Isolation)
ATCC 17978	Meningitis, ATCC, USA
ATCC 19606	Urine, ATCC, USA
ATCC 19606 (R)	LPS mutant [55]
Pk.A1	Sepsis, Blood, Pakistan, 2023
Pk.A2	Sepsis, Blood, Pakistan, 2023
Pk.A3	Sepsis, Blood, Pakistan, 2023
Pk.A5	Sepsis, Blood, Pakistan, 2023
Pk.A6	Sepsis, Blood, Pakistan, 2023
Pk.A8	Sepsis, Blood, Pakistan, 2023
Pk.M1	Sepsis, Blood, Pakistan, 2023
Pk.M2	Sepsis, Blood, Pakistan, 2023
Pk.M3	Sepsis, Blood, Pakistan, 2023
Pk.M4	Sepsis, Blood, Pakistan, 2023
Pk.M5	Sepsis, Blood, Pakistan, 2023
PkM8	Sepsis, Blood, Pakistan, 2023
Pk.M9	Sepsis, Blood, Pakistan, 2023
Pk.M10	Sepsis, Blood, Pakistan, 2023
Pk.M11	Sepsis, Blood, Pakistan, 2023
PW01a-AA1	Unknown origin, Australia, 2012
PW01b-AA2	Unknown origin, Australia, 2012
PW01c-AA6	Unknown origin, Australia, 2012
RB01-AA7	Bronchiectasis, Sputum, Australia, 2012
RB02d1-AA8	Tracheal aspirate, Australia, 2012
WM00-AA3	Bronchiectasis, Sputum, Australia, 2012
WM97a-AA9	Bronchiectasis, Sputum, Australia, 2012
WM98-AA4	Wound, Australia, 2012
WM99c-AA5	Sputum, Australia, 2012
Ab08	Inguinal swab, Switzerland, 2014
Ab39	Rectal swab, Switzerland, 2020
Ab61	Pharyngeal swab, Switzerland, 2016

Color code: Light Gray = ATCC strains; Green = Pakistani isolates; Yellow = Australian isolates; Red = Swiss isolates.

**Table 2:** Minimum inhibitory concentration (MIC) of different cationic antimicrobials against *Acinetobacter baumannii* isolates.

Strain	MIC (μg/mL)		MIC (μg/mL)						
	S ≤ 2, I = 4, R ≥ 8 *								
	Polymyxin B	Colistin	Mel4	Melimine	LL-37	LFcin	PQ-1	PHMB	CHX
ATCC 17978	2	0.5	15.6	62.5	62.5	>500	25	12.5	25
ATCC 19606	1	0.5	62.5	62.5	62.5	>500	25	12.5	25
ATCC 19606 (R)	1250	>2500	62.5	62.5	62.5	>500	12.5	6.3	6.3
Pk.A1	1	1	250	250	62.5	ND	12.5	25	12.5
Pk.A2	2	2	250	250	31.3	ND	6.3	25	50
Pk.A3	2	1	250	250	62.5	ND	12.5	50	12.5
Pk.A5	1	1	15.6	250	62.5	ND	25	25	50
Pk.A6	1	0.5	250	250	62.5	ND	25	25	50
Pk.A8	2	1	250	250	62.5	ND	50	12.5	25
Pk.M1	2	1	250	125	62.5	ND	12.5	25	25
Pk.M2	1	1	250	125	31.3	ND	25	12.5	6.3
Pk.M3	2	2	250	125	62.5	ND	25	6.3	25
Pk.M4	1	2	250	125	62.5	ND	12.5	12.5	25
Pk.M5	1	2	250	125	62.5	ND	12.5	12.5	12.5
Pk.M8	1	2	250	125	31.3	ND	12.5	25	12.5
Pk.M9	1	1	250	125	62.5	ND	25	12.5	12.5
Pk.M10	1	1	62.5	62.5	62.5	ND	6.3	25	6.3
Pk.M11	1	2	125	250	62.5	ND	6.3	25	25
PW01a-AA1	1	1	125	62.5	62.5	>500	50	25	25
PW01b-AA2	1	1	125	62.5	62.5	>500	12.5	50	25
WM00-AA3	2	1	125	125	62.5	>500	25	25	25
WM98-AA4	1	0.5	62.5	62.5	62.5	>500	25	25	25
WM99c-AA5	2	1	15.6	125	125	>500	25	25	25
PW01c-AA6	1	0.5	125	125	125	>500	12.5	25	25
RB01-AA7	1	0.5	31.3	250	62.5	>500	50	50	50
RB02d1-AA8	1	0.25	15.6	125	125	>500	50	12.5	25
WM97a-AA9	2	1	15.6	62.5	125	>500	25	6.25	25
Ab08	16	8	62.5	62.5	62.5	ND	6.3	3.1	25
Ab39	32	16	250	125	125	ND	6.3	3.1	50
Ab61	64	64	500	125	125	ND	6.3	3.1	6.3

* R = resistant, I = intermediate and S = Sensitive, cut offs for polymyxin B and Colistin from CLSI for *A. baumannii*; Lfcin = lactoferricin; PQ-1 = polyquaternium-1; PHMB = polyhexamethylene biguanide; CHX = chlorhexidine. ND = not done; Color code: Light Gray = ATCC strains; Green = Pakistani isolates; Yellow = Australian isolates; Red = Swiss isolates.

## Data Availability

Data are contained in the article.

## Abbreviations

The following abbreviations are used in this manuscript:

Lfcin	Lactoferricin
PQ-1	polyquaternium-1
PHMB	polyhexamethylene biguanide
CHX	chlorhexidine
